# IL-33 Aggravates DSS-Induced Acute Colitis in Mouse Colon Lamina Propria by Enhancing Th2 Cell Responses

**DOI:** 10.1155/2015/913041

**Published:** 2015-05-28

**Authors:** Junfeng Zhu, Fangli Yang, Lixuan Sang, Jingbo Zhai, Xiaoqing Zhang, Dan Yue, Shengjun Li, Yan Li, Changlong Lu, Xun Sun

**Affiliations:** ^1^Department of Immunology, China Medical University, Shenyang 110122, China; ^2^School of Life Science, Liaoning University, Shenyang 110036, China

## Abstract

Interleukin- (IL-) 33, a member of the IL-1 cytokine family, is an important modulator of the immune system associated with several immune-mediated diseases. IL-33 was expressed in high level on epithelial cells of intestinal tract. It suggested that IL-33 plays a potential role in inflammatory bowel diseases (IBD). We investigated the role of interleukin- (IL-) 33 in dextran sulphate sodium- (DSS-) induced acute colitis in mice using recombinant mouse IL-33 protein (rIL-33). We found that DSS-induced acute colitis was aggravated by rIL-33 treatment. rIL-33-treated DSS mice showed markedly reduced levels of interferon- (IFN-)*γ* and IL-17A in their colon lamina propria lymphocytes (LPL), but the levels of Th2 cytokines, such as IL-5 and IL-13, in these cells were significantly increased, compared to DSS mice treated with PBS. Our results suggested that IL-33 stimulated CD4^+^T cells and caused the cell to adopt a Th2-type response but at the same time suppressed Th17 and Th1 cell responses. Therefore, IL-33 may be involved in pathogenesis of DSS-induced acute colitis by promoting Th2 cell response in intestinal mucosa of mice. Modulation of IL-33/ST2 signaling by monoclonal antibody (mAb) could be a novel biological therapy in DSS-induced acute colitis.

## 1. Introduction

Inflammatory bowel diseases (IBD), including Crohn's disease (CD) and ulcerative colitis (UC), are characterized by inflammation in the large and/or small intestine associated with uncontrolled innate and adaptive immunity against normal constituents, including commensal bacteria and various microbial products [1, 2]. We used to think that the responding T cells exhibit a T-helper (Th) cell type 1 phenotype capable of producing IFN-*γ* in CD [3], whereas Th2 cytokine, such as IL-4, IL-5, or IL-13, is closely associated with UC [4]. In addition to these major immune responses associated with CD and UC, Th17, which mainly produces IL-17A, has also been linked to pathogenesis of IBD recently [5, 6]. Among various experimentally induced colitis models in mice, dextran sulphate sodium- (DSS-) induced acute or chronic colitis is the most commonly used as IBD animal models [7, 8]. In the DSS-induced acute colitis model, mice are treated with DSS polymers in the drinking water and this colitis model is characterized by diarrhea, bloody faeces, weight loss, and a histological picture of inflammation and ulceration as seen in UC [9, 10]. Although the acute intestinal inflammation can be induced by DSS in the T cell-independent condition [10], the adaptive immune responses mediated by Th2 cell play a needful role in the pathogenesis of DSS-induced acute colitis [11].

Similar to IL-1 and IL-18, IL-33, also known as NF-HEV, is a newly identified cytokine belonging to the IL-1 family that is mainly expressed in endothelial cells of all organs and in epithelial cells of mucosa-associated tissues such as the intestine and airways [12–16]. ST2 is the IL-33 receptor and exists in two different splice variants leading to the synthesis of ST2L, a transmembrane receptor that confers the biologic effects on IL-33 [17, 18], and sST2, a soluble molecule which likely serves as a decoy receptor by interacting with IL-33 and blocking its biological effects [19, 20]. As recent reports, ST2L is mainly expressed on Th2 cells [12, 21, 22]. Therefore, IL-33/ST2 axis has been shown to play an important role in intestinal inflammation associated with a predominant Th2 response.

Although the levels of IL-33 are elevated in human UC [23, 24], the role of increased IL-33 production during intestine inflammation remains unclear. There are several lines of evidence showing that IL-33 plays a pathogenic role in acute intestinal inflammation [25–28]. However, a number of recent studies have suggested that IL-33 attenuates the development and perpetuation of chronic intestinal inflammation [25, 29]. These findings have also been replicated in the trinitrobenzene-sulfonic-acid- (TNBS-) induced colitis model [30]. There are several lines of evidence showing that the IL-33 is associated with the development of Th2 immunity, and it induces the production of Th2-type cytokines [27, 30]. However, little is known about the role of IL-33 in UC. In the present study, we used DSS-induced acute colitis (a representative colitis model for UC) in mice to elucidate the influence of IL-33 in the development of inflammatory condition.

## 2. Materials and Method

### 2.1. Animals

Seven-week-old male C57BL/6 mice weighing about 18–22 g were purchased from Beijing HFK Bioscience Co., Ltd. (Beijing, China). The mice were maintained under special-pathogen-free conditions at China Medical University for at least 1 week before being used in experiments. All of the studies were performed in accordance with the China Medical University Animal Care and Use Committee guidelines.

### 2.2. Expression and Purification of rIL-33

Full-length mouse IL-33 was amplified from mouse spleen cDNA and cloned into a pUC-T vetor (Beijing CoWin Biotech, Beijing, China). The insert was confirmed by direct DNA sequencing. The cDNA sequence, starting with amino acid 112 of the full-length protein, was then amplified from the above plasmid containing the mature IL-33 using specific primer pairs: 5′-GCATGAATTCATGACATTGAGCATCCAAGGAAC-3′ (forward) and 5′-CCGCCTCGAGGATTTTCGAGAGCTTAAACA-3′ (reverse). The resulting amplified fragment was inserted into the expression vector pET21a (+) at the* EcoR* I and* Xho* I to yield the construct pET21a-IL-33. This construct was transformed into* Escherichia coli* strain BL21DE3, and the expression of rIL-33 was induced by isopropyl-*β*-d-thiogalactoside and purified by using 6 × His-Tagged Protein Purification Kit (Beijing CoWin Biotech, Beijing, China), followed by ToxinEraser Endotoxin Removal Kit (GenScript, Nanjing, China) to remove any endotoxin that might have come from the host cells. The purity of rIL-33 was more than 95% tested by SDS-PAGE, and the endotoxin levels were less than 1 Eu/mg of protein using ToxinSensor Chromogenic LAL Endotoxin Assay Kit (GenScript, Nanjing, China).

### 2.3. Antibodies and Reagents

Antibodies for flow cytometry analysis, Fc*γ* receptor-blocking mAb (CD16/32; 2.4G2), anti-Gr-1 (RB6-8C5), anti-CD4 (RM4-5), anti-CD44 (IM7), anti-IL-17A (TC11-18H10.1), and anti-IFN-*γ* (XMG1.2) were purchased from BD Biosciences (San Diego, CA, USA). Purified anti-CD3 and anti-CD28 were also obtained from BD Biosciences (San Diego, CA, USA). Anti-F4/80 (BM8), anti-B220 (RA3-6B2), anti-TCR*β* (H57-597), anti-CD8 (53–6.7), anti-NK1.1 (PK136), anti-TCR*γδ* (GL3), and anti-CD25 (PC61) were purchased from Biolegend (San Diego, CA, USA).

### 2.4. Induction of Acute Colitis Model

To induce acute colitis in mice, the animals were orally administered 2.5% DSS (MW 36,000–50,000, MP Biomedicals, USA) for 7 days. Mice were treated with rIL-33 (1 *μ*g in 100 *μ*L PBS/mouse/day) or PBS (100 *μ*L/mouse/day) as control on every day. Body weight and the disease activity index (DAI) were monitored daily. DAI was determined by scoring body weight loss, trait of stool, and occult blood in stool or hematochezia according to the classic scoring system described by Cooper et al. [31]. The scoring process is given as follows: body weight loss (0, none; 1, 1%–5%; 2, 5%–10%; 3, 10%–20%; 4, >20%), stool consistency (0, normal; 2, loose stool; 4, diarrhea), and stool blood (0, negative; 2, fecal occult blood test positive; 4, gross bleeding). All the animals were sacrificed at the end of the experiment, and the colon tissue was removed and cleaned and then subjected to cell culture, flow cytometry, quantitative real-time PCR, and histological analyses.

### 2.5. Histological Assessment of Colitis

The middle part of colon was fixed with 4% paraformaldehyde, and the fixed tissue was then embedded in paraffin. Five-micrometer tissue sections were sliced and stained with H&E. Histology was scored as described previously [32]. Histology was scored as follows: epithelium (E): 0, normal morphology; 1, loss of goblet cells; 2, loss of goblet cells in large areas; 3, loss of crypts; 4, loss of crypts in large areas and infiltration (I): 0, no infiltrate; 1, infiltrate around the crypt basis; 2, infiltrate reaching the L muscularis mucosae; 3, extensive infiltration reaching the L muscularis mucosae and thickening of the mucosa with abundant edema; 4, infiltration of the L submucosa. The total histologic score was given as E + I.

### 2.6. Cell Preparation

Lamina propria (LP) cells in the colon were isolated by a modified method described previously [33]. In brief, gut pieces were cut into 2-mm slices and the epithelium was eliminated by stirring, first in PBS containing 3 mM EDTA for 10 min at 37°C (twice) and then in RPMI (Sigma Chemical Co., St. Louis, MO, USA) containing 1% FBS, 1 mM EGTA, and 1.5 mM MgCl_2_ for 15 min (also twice). Gut pieces were collected and stirred in RPMI containing 20% FBS, 100 U/mL collagenase (C2139; Sigma-Aldrich Corp., St. Louis, MO, USA), and 5 U/mL DNase 1 (Sigma-Aldrich Corp) for 90 min at 37°C. Halfway through the incubation and at the end of the incubation, the suspension was dissociated by multiple aspirations through a syringe for 2 min. The pellet was purified to LPL on a 45%/66.6% discontinuous Percoll (Pharmacia, Uppsala, Sweden) gradient at 600 ×g for 20 min. The number of viable cells was counted by trypan blue staining.

### 2.7. Enzyme-Linked Immunosorbent Assay (ELISA)

To measure spontaneous cytokine production by LPL, LPL were cultured in 96-well flat-bottom plates without any stimulation for 24 h at 37°C under 5% CO_2_. The cells were cultured in 0.2 mL RPMI 1640 (Wako, Osaka, Japan) containing 10% FBS (Cell Culture Technologies, Tokyo, Japan). The production of TNF-*α*, IL-6, IL-1*β*, IL-23, or IL-12p70 was measured using ELISA kits (R&D Systems). Isolated LPL were incubated for 48 h* in vitro* in 96-well flat-bottom plates (Falcon; BD Biosciences) coated with anti-CD3 (10 *μ*g/mL) and anti-CD28 (1 *μ*g/mL) antibodies. IFN-*γ*, IL-17A, IL-4, IL-10, IL-5, and IL-13 secreted into the culture medium were measured with ELISA kits (R&D Systems) according to the manufacturer's instruction.

### 2.8. Real-Time Quantitative Polymerase Chain Reaction (RT-PCR)

Total RNA was extracted from colon of mice using the RNAiso plus (Takara, Dalian, China) according to the manufacturer's instruction. 1 *μ*g of total RNA from the colon of each mouse was used for reverse transcription performing using a PrimeScript RT reagent kit with gDNA Eraser (Perfect Real Time, Takara) to generate the first strand cDNA. PCR mixture was prepared using SYBR Premix Ex Taq (Tli RNaseH Plus, Takara) and one of primers listed in Table 1. The amplification program consisted of the following steps: 95°C for 30 sec, and 40 cycles of 95°C for 15 sec and 60°C for 34 sec on an ABI PRISM 7500 Sequence Detection System (Applied Biosystems, Foster City, CA). Each gene expression was normalized with *β*-actin mRNA content.

### 2.9. Flow Cytometry

Isolated LPL were incubated with an FcgR-blocking mAb and stained with mAbs against mouse Gr-1, F4/80, *αβ*TCR, *γδ*TCR, NK1.1, CD4, CD44, CD25, and B220. For intracellular cytokine staining, LPL were stimulated with PMA (25 ng/mL; Sigma-Aldrich, St. Louis, MO) and ionomycin (1 *μ*g/mL; Sigma-Aldrich) for 5 h at 37°C. Brefeldin A (10 *μ*g/mL; Sigma-Aldrich) was added after the first hour of incubation. These cells were harvested, washed, and stained with mAbs against mouse IL-17A or IFN-*γ* for 30 min at 4°C. The intracellular expression of IL-17A and IFN-*γ* in CD4^+^ T-LPL was analyzed by flow cytometry using a Cytofix/Cytoperm Kit Plus (BD Biosciences, San Jose, CA) according to the manufacturer's instructions.

### 2.10. Statistical Analysis

The difference in survival rates was assessed by the log rank test (Mantel-Cox). Differences in parametric data were evaluated by a Student's *t*-test. Statistically significant differences were accepted when *P* < 0.05.

## 3. Results

### 3.1. rIL-33 Aggravates DSS-Induced Acute Intestinal Inflammation in Mice

**Figure 1:**
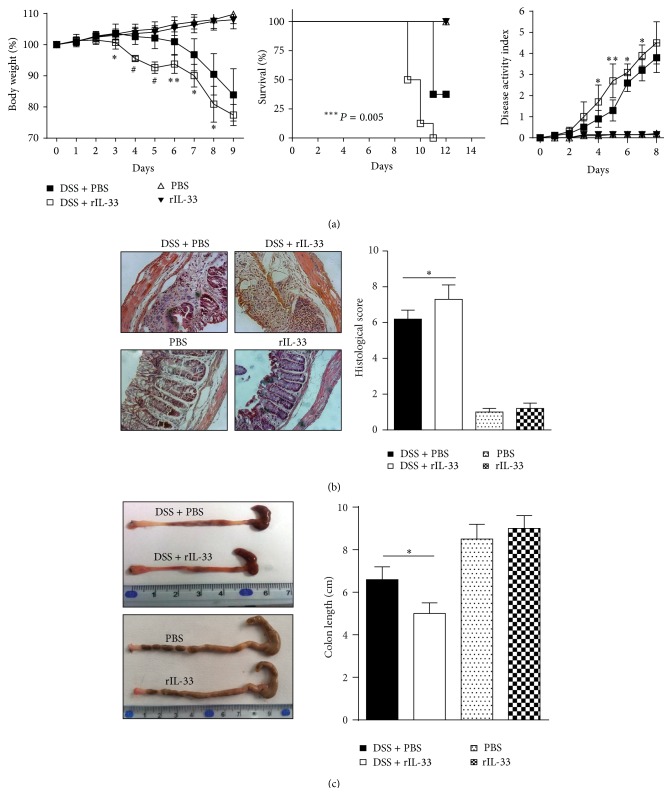
Administration of rIL-33 results in exacerbation of DSS-induced acute colitis. (a) Weight loss, survival rate, and disease activity index; (b) histological score (original magnification, ×200); (c) macroscopic changes and colon length. Data indicate mean ± SD of 8 mice of each group obtained from a representative of three independent experiments (*n* = 8 per group). ^∗^ *P* < 0.05, ^∗∗^ *P* < 0.01, and ^#^ *P* < 0.0001.

Compared to PBS-treated DSS mice, rIL-33-treated DSS mice showed enhanced development of DSS-induced acute colitis as indicated by weight loss, DAI, survival rate, macroscopic changes, and histological score, but there was no significant difference between rIL-33- and PBS-treated normal mice (Figure 1). These results demonstrated that rIL-33 could aggravate the symptoms of DSS-induced acute colitis in mice.

### 3.2. Cell Accumulation in LP of Colon Mucosa in rIL-33-Treated Mice during DSS-Induced Acute Colitis

**Figure 2:**
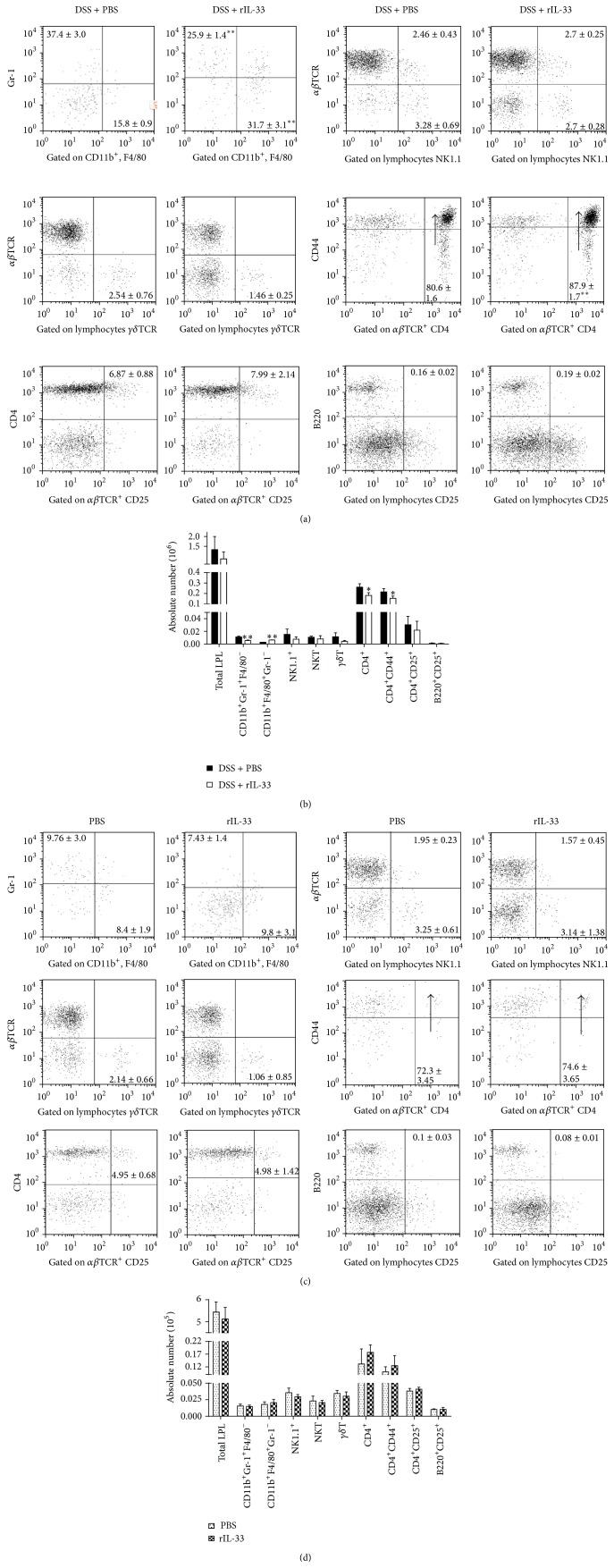
Flow cytometry analysis of the populations of LPL in the colon in rIL-33- and PBS-treated mice with or without acute colitis. ((a), (c)) The percentage of neutrophil, macrophage, CD4^+^, CD4^+^CD44^+^, CD4^+^CD25^+^, B220^+^CD25^+^, *γδ*T cell, and NK and NKT cell in LP in the colon of rIL-33- and PBS-treated mice with or without acute colitis; ((b), (d)) the absolute number of LPL in the colon of rIL-33- and PBS-treated mice with or without acute colitis. Data indicating mean ± SD obtained from a representative of three independent experiments were valued by a Student's *t*-test (*n* = 3 per group). ^∗^ *P* < 0.05; ^∗∗^ *P* < 0.01.

As shown in Figure 2(a), the percentage of macrophage (CD11b^+^F4/80^+^Gr-1^−^) in LPL markedly increased, but the percentage of neutrophil (CD11b^+^Gr-1^+^F4/80^−^) in LPL significantly decreased in colon of mice treated with rIL-33. The absolute numbers of macrophage markedly increased and the absolute numbers of neutrophil significantly decreased in mice treated with rIL-33 compared to PBS controls (Figure 2(b)). The percentage of CD4^+^CD44^+^ cells in CD4^+^T cells markedly increased in colon of rIL-33-treated mice as compared with those in control mice. However, the absolute numbers of CD4^+^ and CD4^+^CD44^+^ T-LPL significantly decreased in rIL-33-treated mice when compared with those in control mice. There were no significant differences in the percent and the absolute numbers of NKT cell (NK1.1^+^ *αβ*TCR^+^), *γδ*T cell (*γδ*TCR^+^ *αβ*TCR^−^), NK cell (NK1.1^+^ *αβ*TCR^−^), CD4^+^CD25^+^T cell, and Breg cell (B220^+^CD25^+^) between DSS mice treated with rIL-33 and PBS. However, there was no significant difference in the cell accumulation in LP of colon between PBS- and rIL-33-treated normal mice (Figures 2(c) and 2(d)).

### 3.3. Cytokine Production by CD4^+^ T-LPL of Colon in Mice Treated with rIL-33 during DSS-Induced Acute Colitis

**Figure 3:**
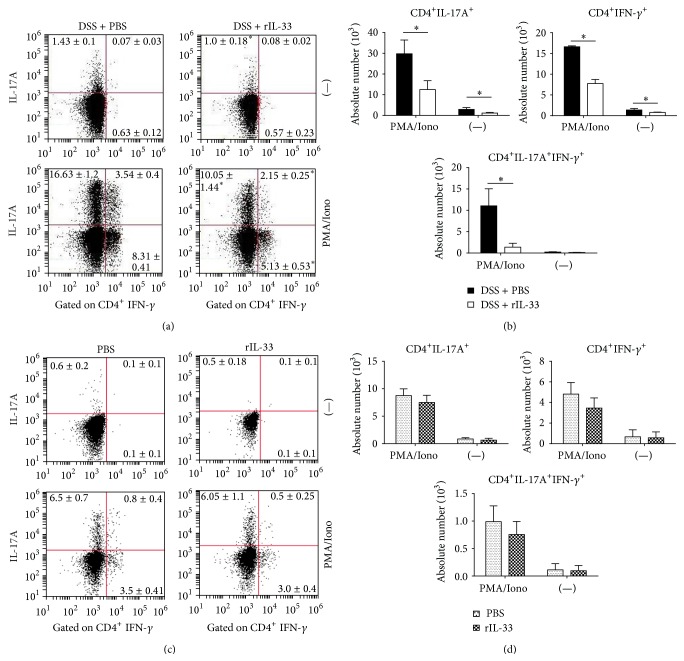
Cytokine-producing T cells in the LPL of colon in rIL-33- and PBS-treated mice with or without DSS-induced acute colitis. ((a), (c)) The percent of CD4^+^IFN-*γ* ^+^ or CD4^+^IL-17A^+^ T-LPL in the LPL of colon in rIL-33- and PBS-treated mice with or without DSS-induced acute colitis. ((b), (d)) The absolute number of CD4^+^IFN-*γ* ^+^ or CD4^+^IL-17A^+^ T-LPL in the LPL of colon in rIL-33- and PBS-treated mice with or without DSS-induced acute colitis. Data indicating mean ± SD obtained from a representative of three independent experiments were valued by a Student's *t*-test (*n* = 4 per group). ^∗^ *P* < 0.05; ^∗∗^ *P* < 0.01.

We further examined the intracellular cytokine production by CD4^+^ T-LPL of colon in rIL-33- or PBS-treated DSS mice. Consistent with the cell profile observed above, there was a significant reduction in the frequency of CD4^+^IL-17A^+^ and CD4^+^IFN-*γ* ^+^ cells in LPL of rIL-33-treated DSS mice (Figure 3(a)). Furthermore, we observed a reduction in the frequency of CD4^+^IL-17A^+^IFN-*γ* ^+^ cells in LPL from rIL-33-treated DSS mice (Figure 3(a)) with a marked reduction in the absolute number of these cells (Figure 3(b)). However, there was a reduction but no significant difference in the frequency or the absolute number of CD4^+^IL-17A^+^ and CD4^+^IFN-*γ* ^+^ cells in LPL between rIL-33- and PBS-treated normal mice (Figures 3(c) and 3(d)).

### 3.4. rIL-33 Shifts the Cytokine and Transcription Factors Production of DSS-Induced Acute Colitis

**Figure 4:**
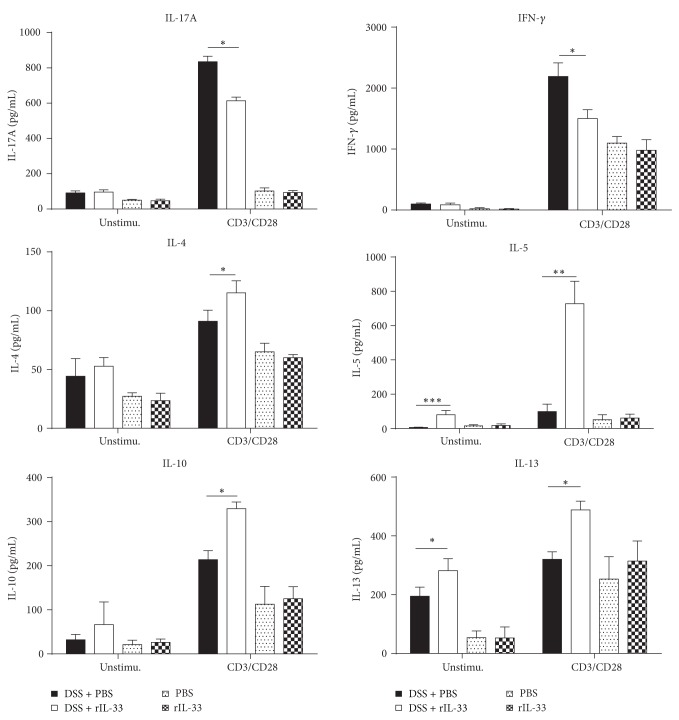
ELISA analysis of cytokine production in LP of colon in rIL-33- and PBS-treated mice with or without DSS-induced acute colitis. The level of IFN-*γ*, IL-17A, IL-4, IL-5, IL-13, or IL-10 production in the culture supernatants of LPL of colon was analyzed by ELISA with or without anti-CD28/anti-CD3 mAbs stimulations. Data indicating mean ± SD obtained from a representative of three independent experiments were valued by a Student's *t*-test (*n* = 4 per group). ^∗^ *P* < 0.05; ^∗∗^ *P* < 0.01; ^∗∗∗^ *P* < 0.001.

**Figure 5:**
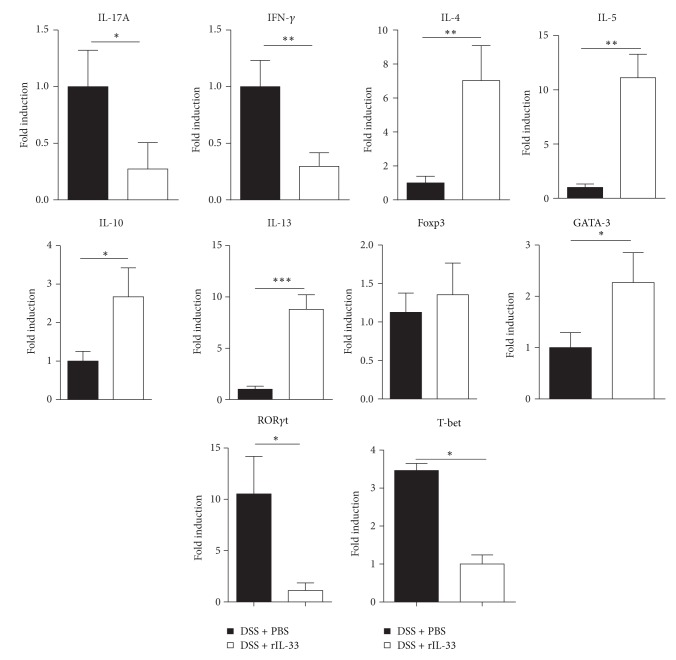
Total mRNA was extracted from colonic tissues to analyze the expression of IL-4, IL-5, IL-13, IFN-*γ*, IL-17A, IL-10, Foxp3, T-bet, GATA-3, and ROR*γ*t by real-time PCR. Data indicating mean ± SD obtained from a representative of three independent experiments were valued by a Student's *t*-test (*n* = 5 per group). ^∗^ *P* < 0.05; ^∗∗^ *P* < 0.01; ^∗∗∗^ *P* < 0.001.

To explore whether rIL-33-induced alleviation of DSS-induced acute colitis correlated with T-cell immunity, we analyzed the levels of cytokines of LPL. In the case of rIL-33-treated DSS mice, a marked reduction in the expression of IFN-*γ* and IL-17A and increased production of Th2-type cytokines (IL-4, IL-5, IL-10, and IL-13) were observed, compared to PBS-treated DSS mice (Figure 4). Similar results were also observed by real-time PCR analysis (Figure 5). rIL-33 administration leads to a reduction in expression of T-bet and ROR*γ*t, but it leads to an increase in expression of GATA-3 (Figure 5). Other cytokines (TNF-*α*, IL-1*β*, IL-6, IL-23, and IL-12p70) were detected in low levels and were not significantly different between the rIL-33- and PBS-treated mice with DSS colitis (data not shown). Nonetheless, there was no significant difference in the production of IL17A, IFN-*γ*, IL-4, IL-5, IL-10, or IL-13 between rIL-33- and PBS-treated normal mice. These results suggested that rIL-33 treatment of mice who underwent DSS-induced acute colitis couldpromote Th2 immunity.

## 4. Discussion

In the present study, we examined the role of rIL-33 in DSS-induced acute colitis in mice. We found that rIL-33 may be involved in the development of DSS-induced acute colitis by promoting Th2 cell response in LP of the colon. Our results suggested that modulation of IL-33/ST2 signaling by mAb could be a novel therapy for UC.

The Th1/Th2 paradigm was used to differentiate the underlying immunological conditions of CD and UC. The dominant paradigm was that CD was characterized by a Th1 mucosal immune response, with overproduction of IFN-*γ*, while UC was thought to be characterized by a Th2 response, with excess production of IL-5 and IL-13 [34, 35]. DSS-induced acute colitis is known as a UC model, and many studies have described UC as a Th2 disease [36, 37]. Recently, Th17 has also been involved in the development of IBD [5, 6].

In this study, we found that rIL-33 shows the aggravating role in DSS-induced acute colitis, an inflammation model that mimics UC driven by Th2-type immune mechanisms. Furthermore, we show that the exacerbation effect of rIL-33 is accompanied by suppressing the Th1 and Th17 responses but improving Th2 reponses.

Exacerbation of DSS-induced acute colitis, characterized by Th2 cytokine response, indicated that rIL-33 may influence processes during DSS-induced acute colitis in several ways. First, rIL-33 is capable of specifically inducing increased production of the key pathogenic Th2-type cytokines IL-4, IL-5, and IL-13. In a recent study, rIL-33 induced Th2-type cytokines directly via IL-4 and IL-4R in colitis [27]. Moreover, rIL-33 can promote GATA-3 expression (Figure 5), which was consistent with a recent study in which mesenteric lymph node (MLN) T cells stimulated with rIL-33* in vitro* had increased GATA-3 expression [38]. It may be another reason that rIL-33 promotes Th2 cells responses. Second, IL-33 may function as a novel epithelial “alarmin,” which initiates early inflammatory immune responses [13]. This exacerbation of inflammation by rIL-33 administration could be the same as boosting the alarmin-function of endogenous IL-33, which can be released by damaged epithelial cells. IL-33 can act on diverse innate immune cells (mast cells [39], eosinophils [40], basophils [41], DCs [22], macrophages [42], and neutrophils [43]) and therefore contributes to initiation of inflammation. Third, IL-33 may induce type 2 innate lymphoid cells (ILC2s) to produce IL-5 and IL-13. ILC2s, expressing significantly greater levels of ST2 and CD25 [44], need GATA-3 for their development [45, 46] and produced type 2 cytokines, such as IL-5 and IL-13 [47]. In our study, rIL-33 can promote IL-5 and IL-13 production without any stimulation and expression of GATA-3 (Figures 4 and 5). So, one possibility of the exacerbation of DSS-induced acute colitis by rIL-33 treatment may be that rIL-33 induced ILC2s to produce type 2 cytokines.

IFN-*γ* and IL-17A are the key cytokines for Th1 and Th17 responses and are also thought to play pathogenic roles in CD and the chronic stage of DSS-induced colitis [48–50]. Recent papers reported that IL-33 attenuates EAE and experimental autoimmune uveitis by suppressing IL-17A and IFN-*γ* production [51, 52]. Similar results have also been found in our study (Figures 3–5). These results suggested that IL-33 might have a therapeutic effect in CD and the other Th1/Th17-mediated immune response and mucosal inflammation.

## 5. Conclusion

The results of this study demonstrate that rIL-33 administered has an aggravated role in DSS-induced acute colitis possibly through the induction of Th2-type cytokines (IL-5 and IL-13) production together with suppression of Th1/Th17 response, and thus, by extension, modulation of IL-33/ST2 signaling by mAb may be a potential therapeutic reagent for human UC. However, the exact function of IL-33 in the IBD remains unclear and merits further investigation.

## Figures and Tables

**Table 1:** Primer sequences for RT-PCR.

Gene	Forward	Reverse
IL-4	5′-CATCGGCATTTTGAACGAG-3′	5′-TTGGAAGCCCTACAGACGAG-3′
IL-5	5′-AGCACAGTGGTGAAAGAGAC-3′	5′-TCCAATGCATAGCTGGTGATTT-3′
IL-10	5′-GGTTGCCAAGCCTTATCGGA-3′	5′-ACCTGCTCCACTGCCTTGCT-3′
IL-13	5′-GGAGCTGAGCAACATCACAC-3′	5′-GGTCCTGTAGATGGCATTGCA-3′
IL-17A	5′-GCTCCAGAAGGCCCTCAGA-3′	5′-AGCTTTCCCTCCGCATTGA-3′
IFN-*γ*	5′-AAAGACAATCAGGCCATCAG-3′	5′-TGGGTTGTTGACCTCAAACT-3′
Foxp3	5′-GGCCCTTCTCCAGGACAGA-3′	5′-GCTGATCATGGCTGGGTTGT-3′
GATA-3	5′-ACAGCTCTGGACTCTTCCCA-3′	5′-GTTCACACACTCCCTGCCTT-3′
T-bet	5′-CCAGGGAACCGCTTATATGT-3′	5′-CTGGGTCACATTGTTGGAAG-3′
ROR-*γ*t	5′-CCACTGCATTCCCAGTTTCT-3′	5′-CGTAGAAGGTCCTCCAGTCG-3′
*β*-actin	5′-TTCCAGCGTTCCTTCTTGGGT-3′	5′-GTTGGCATAGAGGTGTTTACG-3′
